# Mitochondrial and Y-chromosomal profile of the Kazakh population from East Kazakhstan

**DOI:** 10.3325/cmj.2013.54.17

**Published:** 2013-02

**Authors:** Pavel V. Tarlykov, Elena V. Zholdybayeva, Ainur R. Akilzhanova, Zhannur M. Nurkina, Zhaxylyk M. Sabitov, Tolebay K. Rakhypbekov, Erlan M. Ramanculov

**Affiliations:** 1 National Center for Biotechnology of the Republic of Kazakhstan, Astana, Kazakhstan; 2 L.N. Gumilyov Eurasian National University, Astana, Kazakhstan; 3 Semey State Medical University, Semey, Kazakhstan

## Abstract

**Aim:**

To study the genetic relationship of Kazakhs from East Kazakhstan to other Eurasian populations by examining paternal and maternal DNA lineages.

**Methods:**

Whole blood samples were collected in 2010 from 160 unrelated healthy Kazakhs residing in East Kazakhstan. Genomic DNA was extracted with Wizard® genomic DNA Purification Kit. Nucleotide sequence of hypervariable segment I of mitochondrial DNA (mtDNA) was determined and analyzed. Seventeen Y-short tandem repeat (STR) loci were studied in 67 samples with the AmpFiSTR Y-filer PCR Amplification Kit. In addition, mtDNA data for 2701 individuals and Y-STR data for 677 individuals were retrieved from the literature for comparison.

**Results:**

There was a high degree of genetic differentiation on the level of mitochondrial DNA. The majority of maternal lineages belonged to haplogroups common in Central Asia. In contrast, Y-STR data showed very low genetic diversity, with the relative frequency of the predominant haplotype of 0.612.

**Conclusion:**

The results revealed different migration patterns in the population sample, showing there had been more migration among women. mtDNA genetic diversity in this population was equivalent to that in other Central Asian populations. Genetic evidence suggests the existence of a single paternal founder lineage in the population of East Kazakhstan, which is consistent with verbal genealogical data of the local tribes.

In terms of population genetics, Central Asia is one of the least studied regions in the world. The studies conducted in the region, based on scarce genetic data, indicate that the Central Asia population is a mix of Eastern and Western populations (1,2). Kazakhstan is a vast country, which has throughout history been inhabited by different nomadic tribes such as the Argyn, Dughlat, Jalayir, Kerei, Kipchak, Madjar, Naiman, and others (3). The Kazakh ethnic group was formed in the 15th century under a huge infulence of the Mongol Empire (4). We expected the genetic profile of Kazakhs to be heterogeneous because of the different tribes and ethnicities (5).

The current study focused on the Kazakh population of the East Kazakhstan Province, because recently there have been many reports on the neighboring populations of Xinjiang Uyghur Autonomous Region and Altai regions. East Kazakhstan is populated by the Naiman tribe. Their genealogical narrative, “*shezhire*,” states that the Naiman people living in Tarbagatay region are descendants of one ancestor, named Toktar-kozha, who came from the territory of modern Uzbekistan and was a Sart by origin. Based on the data from “*shezhire,*” we formed a hypothesis of uniform paternal descent of the Naiman tribe. The aim of this study was to better understand the origins and differentiation of the Kazakh ethnic group and to investigate the genetic relationship between this population and other Eurasian populations.

## Materials and methods

### Participants and reference data

A total of 160 blood samples were collected from healthy adult individuals, 67 men and 93 women, during an expedition to Tarbagatay region, East Kazakhstan in 2010. Prior to the expedition, ethical approval was received from the Ethics Committee of the National Center for Biotechnology of the Republic of Kazakhstan (No.10, 14.02.2010). The Ethics Committee approved the informed consent form and questionnaire form designed specifically for the study.

**Figure 1:**
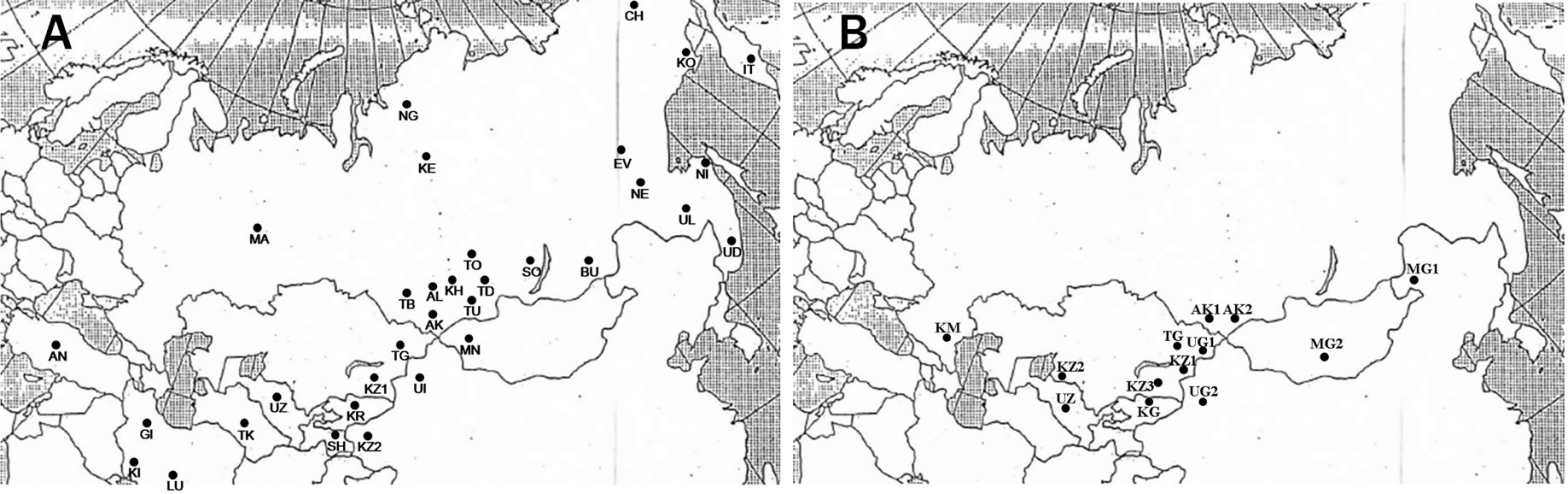
(**A**) Map of Eurasia and locations of samples for the cited mitochondrial DNA studies. The following abbreviations were used to assign locations of population sampling: AK – Altaian Kazakhs (6), KZ1 – Kazakhs (1), KZ2 – Kazakhs (7), KR – Kirghiz (1), MN – Mongolians (6), UI – Uighurs (7), AL – Altaians (8), KH – Khakassians (8), BU – Buryats (8), SO – Sojots (8), TD – Todjins (8), TU – Tuvinians (8), TO – Tofalars (8), MA – Mansi (9), KE – Ket (10), NG – Nganasan (9), TB – Tubalar (11), EV – Evenki (11), NE – Negedal (11), UL – Ulchi (11), NI – Nivkhi (11), UD – Udegey (11), IT – Itelmen (12), KO – Koriak (12), CH – Chukchi (13), AN – Turks (14), GI – Gilaki (14), KI – Kurdish (14), LU – Lur (14), UZ – Uzbek (14), TK – Turkmen (14), SH – Shugnan (14), TG – Kazakhs (this study). (**B**). Map of Eurasia and locations of samples for the cited Y- short tandem repeats (STR) studies The following abbreviations were used to assign locations of population sampling: AK1 – Altaian Kazakhs (15), AK2 – Altaian Kazakhs (15), KM – Kalmyks (16), KZ1 – Kazakhs (17), KZ2 – Kazakhs (18), KG – Kirghiz (17), MG1 – Mongolians (19), MG2 – Mongolians (19), TG – Tarbagatay (this study), UI – Uighurs (17), UI2 – Uighurs (19), UZ – Uzbeks (18), KZ3 – Kazakhs from South Kazakhstan (accession number YA003729, *www.yhrd.com*)

The ethnic origin of sampled individuals was ascertained up to three generations. Blood was taken with the informed consent signed by all donors. In addition, all participants completed the questionnaire that included information on the geographic origin, nationality, maternal and paternal pedigree, and health issues. Related individuals were not included into sampling. In addition, mtDNA haplogroup data for 2701 individuals and Y-short tandem repeat (STR) haplotype data for 677 individuals of different ethnic backgrounds were retrieved from the literature to establish the genetic relationship of Kazakhs from East Kazakhstan with other populations (Supplementary Table 1 (web extra material 1) and Supplementary Table 2 (web extra material 2)) (Figure 1).

**Table 1:** Mitochondrial DNA haplogroup frequencies in the selected populations of Eurasia*

	A (%)	B (%)	C (%)	D (%)	F (%)	G (%)	H (%)	J (%)	K (%)	M (%)	N (%)	T (%)	U (%)	Z (%)	Other (%)
**AK**	4.2	9.3	10.5	17.6	7.2	9.2	10.9	3	0.8	5.4	5.1	1.7	8	0.4	6.7
**KZ1**	9.1	5.4	7.3	18.2	3.6	5.5	21.9	0	0	7.3	0	7.3	5.4	1.8	7.2
**KZ2**	3.8	3.8	13.2	13.2	7.5	3.8	13.1	1.9	0	9.5	0	7.5	3.8	11.3	7.6
**MN**	3.4	2.2	18	31.4	6.7	6.7	5.6	6.7	3.4	7.9	0	2.2	1.1	3.4	1.3
**KR**	4.2	6.4	12.6	20	2.1	8.4	21	5.3	0	6.3	0	1.1	4.3	1.1	7.2
**YU**	7.3	7.3	1.8	16.4	7.3	1.8	10.9	0	3.6	10.9	0	1.8	16.4	0	14.5
**AL**	0	3.6	19.1	15.5	9.1	1.8	6.4	3.6	0	7.3	7.2	0.9	16.4	4.6	4.5
**KH**	3.8	3.8	35.8	13.2	22.6	0	3.8	1.9	0	0	1.9	1.9	11.3	0	0
**BU**	2.2	6.6	28.5	33	1.1	14.3	2.2	2.2	0	3.3	0	1.1	1.1	1.1	3.3
**SO**	10	3.3	20	46.8	0	6.7	0	0	3.3	0	0	0	3.3	0	6.6
**TD**	4.2	4.2	47.6	4.2	2.1	18.8	2.1	0	0	4.2	0	0	6.3	0	6.3
**TU**	1.1	7.8	47.9	17.8	2.2	6.6	1.1	5.6	0	0	1.1	1.1	3.3	1.1	3.3
**TO**	5.2	3.5	62	0	0	1.7	6.9	8.6	0	0	0	5.2	0	5.2	1.7
**MA**	3.1	0	17.3	8.3	1	6.1	14.3	12.2	3.1	1	0	7.2	25.4	0	1
**KE**	7.9	0	15.8	2.6	23.7	0	10.5	0	0	0	0	0	34.2	2.6	2.7
**NG**	0	0	33.3	29.2	0	0	8.4	0	0	0	0	0	24.9	4.2	0
**TB**	11.1	4.2	19.4	19.5	1.4	0	5.6	0	0	0	6.9	0	26.3	1.4	4.2
**EV**	5.6	0	71.9	21.1	1.4	0	0	0	0	0	0	0	0	0	0
**NE**	0	12.1	15.2	24.3	0	27.2	0	0	0	0	0	0	0	0	21.2
**UL**	0	0	13.7	21.9	1.1	11.5	0	0	0	2.3	6.9	0	0	0	42.6
**NI**	0	0	0	28.6	0	5.4	0	0	0	0	0	0	0	0	66
**UD**	0	0	17.4	0	0	0	0	0	0	28.3	30.4	0	0	0	23.9
**IT**	6.4	0	14.9	0	0	68.2	0	0	0	0	0	0	0	6.4	4.1
**CH**	68.2	0	10.6	12.1	0	9.1	0	0	0	0	0	0	0	0	0
**AN**	4	0	0	2	0	2	36	8	6	0	2	8	24	0	8
**GI**	0	0	0	2.7	0	0	37.8	16.3	2.7	2.7	2.7	16.2	18.9	0	0
**KI**	0	0	0	0	0	0	30	10	10	0	0	0	35	0	15
**LU**	0	5.9	0	0	0	0	41.1	5.9	5.9	0	0	5.9	35.3	0	0
**UZ**	7.1	0	2.4	9.4	2.4	2.4	28.6	7.1	0	11.9	7.1	4.8	12	0	4.8
**TK**	2.4	2.4	7.1	22	2.4	0	32.1	9.8	0	4.9	2.4	7.3	4.8	0	2.4
**SH**	2.3	0	18.2	0	0	0	34	4.5	9.2	0	0	2.3	20.4	0	9.1
**KO**	5.2	0	36.1	1.3	0	41.9	0	0	0	0	0	0	0	5.8	9.7
**TG**	8.2	8.8	8.8	34	4.2	3.4	9.5	1.4	0.7	5.4	2	2.7	6.8	1.4	2.7

*Abbreviations: AK – Altaian Kazakhs (6), KZ1 – Kazakhs (1), KZ2 – Kazakhs (7), KR – Kirghiz (1), MN – Mongolians (6), UI – Uighurs (7), AL – Altaians (8), KH – Khakassians (8), BU – Buryats (8), SO – Sojots (8), TD – Todjins (8), TU – Tuvinians (8), TO – Tofalars (8), MA – Mansi (9), KE – Ket (10), NG – Nganasan (9), TB – Tubalar (11), EV – Evenki (11), NE – Negedal (11), UL – Ulchi (11), NI – Nivkhi (11), UD – Udegey (11), IT – Itelmen (12), KO – Koriak (12), CH – Chukchi (13), AN – Turks (14), GI – Gilaki (14), KI – Kurdish (14), LU – Lur (14), UZ – Uzbek (14), TK – Turkmen (14), SH – Shugnan (14), TG – Kazakhs (this study).

**Table 2:** 17-locus Y-short tandem repeat (STR) haplotypes of 67 Kazakhs from East Kazakhstan

	DYS 19	DYS 389I	DYS 389II	DYS 390	DYS 391	DYS 392	DYS 393	DYS 385	DYS 438	DYS 439	DYS 437	DYS 448	DYS 456	DYS 458	DYS 635	YGATA H4
**1-41**	15	12	29	23	10	13	12	13,18	10	12	15	19	15	17	19	12
**42-43**	15	12	29	22	10	10	11	13,18	10	10	14	22	15	16	21	11
**44**	15	15	31	25	10	11	13	13,18	10	11	14	20	15	17	24	10
**45**	15	12	29	23	10	13	12	13,19	10	12	15	19	15	16	19	12
**46**	16	14	31	24	9	11	13	12,16	10	12	14	20	16	17	23	10
**47**	15	13	29	24	10	9	13	12,14	11	11	14	20	17	18	21	11
**48**	15	12	29	24	10	13	13	13,18	10	13	14	23	15	18	22	11
**49**	17	14	32	23	11	11	13	11,14	11	13	14	20	16	16	23	11
**50**	14	14	31	23	11	14	14	11,13	14	10	14	19	13	17	23	11
**51**	14	12	30	23	11	12	10	11,14	12	12	15	19	16	14	24	12
**52**	14	12	30	23	11	12	9	11,14	15	12	15	19	13	14	24	10
**53**	15	11	29	22	9	12	10	13,18	10	11	15	19	15	15	20	11
**54**	15	13	29	25	10	11	13	12,13	12	10	14	22	15	18	21	11
**55**	16	13	30	25	11	11	13	11,14	11	10	14	22	16	15	20	12
**56**	15	12	29	23	10	13	8	13,17	10	12	15	19	15	17	19	12
**57**	15	12	31	21	10	10	15	11,13	10	10	14	21	15	14	21	10
**58**	15	11	29	22	9	12	11	13,18	10	11	15	19	15	15	20	12
**59**	15	12	28	22	9	12	10	12,16	10	10	14	20	18	15	20	11
**60**	15	11	29	22	9	12	10	13,17	10	12	15	19	15	15	20	12
**61**	15	11	29	22	10	12	10	11,13	10	11	15	19	15	15	20	12
**62**	15	12	29	23	9	10	12	13,17	11	10	15	19	15	16	22	11
**63**	15	12	28	23	10	13	14	13,18	10	12	15	19	15	17	19	12
**64**	15	14	29	23	10	7	13	13,18	10	13	14	18	15	18	20	11
**65**	16	13	28	26	10	11	13	13,18	9	10	14	23	15	15	20	11
**66**	15	12	29	23	10	13	12	13,19	10	12	15	19	15	18	19	12
**67**	15	11	31	25	12	13	11	13,18	12	12	17	23	15	18	26	11

### Mitochondrial DNA analysis

Blood collection was performed with EDTA-containing evacuated blood collection tubes (Terumo®, Leuven, Belgium) and blood collecting needles (Terumo®). Blood samples were stored in portable refrigerators until DNA was extracted. DNA extraction and purification was performed with Wizard® genomic DNA Purification Kit (Promega, Madison, WI, USA), according to the manufacturer’s instructions.

*Polymerase chain reaction (PCR) amplification.* Mitochondrial DNA polymorphisms were typed for 160 individuals by using conventional PCR, with primers specific to hypervariable segment I (HVS-I) (20). PCR reactions were carried out in 25-μL reaction volume, containing 3.2 pmol of each primer, 10 ng of genomic DNA, 0.2 units of Taq pol enzyme (Lytech, Moscow, Russia), 200 μM of each dNTP, 1 × PCR buffer, and 2.5 mM MgCl_2_(Lytech). The PCR protocol was: 94°C for 10 minutes, followed by 35 cycles of denaturation at 94°C for one minute, annealing at 55°C for one minute, extension at 72°C for one minute, and a final extension step at 72°C for 7 minutes. PCR products were initially checked in 1.5% agarose gel and sequenced on ABI 3730xl DNA analyzer (Applied Biosystems/Hitachi, Tokyo, Japan).

*Sequence alignment and haplogroup analysis.* The obtained sequences were compared with the revised Cambridge Reference Sequence (rCRS, NC_012920) to find polymorphisms by using SeqScape v 2.6 software (Applied Biosystems, Foster City, CA, USA). Identified HVS-I polymorphisms were used for assignment of mtDNA haplogroups. We also included published data on other Eurasian populations for comparative analysis (Supplementary Table 1 (web extra material 1).

*Data analysis.* Mitochondrial DNA haplogroup frequencies and haplogroup diversity values were calculated as described elsewhere (21). The haplogroups were combined into 15 groups to compare East Kazakhstan data with the published data sets. In case of American populations, only A, B, C, and D mtDNA haplogroup frequency data were used for comparison (Supplementary Table 3 (web extra material 3)). Population pairwise genetic distances were calculated from haplogroup frequencies of the Tarbagatay population and 32 other Eurasian populations with Statistica v.10 software (StatSoft, Tulsa, OK, USA).

**Table 3:** Rst value matrix of Central Asian populations*

	TG	KM	AK1	AK2	KZ1	KG	UI1	KZ2	UZ	UI2	MG1	MG2	KZ3
**TG**	*	<0.001	<0.001	<0.001	<0.001	<0.001	<0.001	<0.001	<0.001	<0.001	<0.001	<0.001	<0.001
**KM**	0.390	*	0.120	<0.001	<0.001	<0.001	<0.001	<0.001	<0.001	<0.001	<0.001	<0.001	<0.001
**AK1**	0.281	0.019	*	0.006	<0.001	<0.001	<0.001	0.382	0.009	<0.001	<0.001	<0.001	0.020
**AK2**	0.259	0.123	0.091	*	<0.001	<0.001	<0.001	<0.001	<0.001	<0.001	<0.001	<0.001	0.001
**KZ1**	0.618	0.654	0.676	0.662	*	0.031	<0.001	<0.001	<0.001	<0.001	<0.001	<0.001	0.000
**KG**	0.467	0.553	0.516	0.518	0.036	*	0.002	<0.001	<0.001	0.007	0.001	<0.001	<0.001
**UI1**	0.246	0.428	0.307	0.369	0.110	0.075	*	<0.001	<0.001	0.866	0.035	0.246	<0.001
**KZ2**	0.070	0.028	-0.003	0.041	0.106	0.080	0.098	*	0.013	<0.001	<0.001	<0.001	<0.001
**UZ**	0.221	0.151	0.085	0.103	0.610	0.437	0.284	0.013	*	<0.001	<0.001	<0.001	<0.001
**UI2**	0.358	0.514	0.432	0.478	0.128	0.072	-0.021	0.080	0.385	*	0.083	0.870	<0.001
**MG1**	0.446	0.584	0.544	0.553	0.174	0.101	0.036	0.103	0.450	0.029	*	0.106	<0.001
**MG2**	0.442	0.572	0.549	0.538	0.151	0.095	0.007	0.124	0.475	-0.016	0.018	*	<0.001
**KZ3**	0.357	0.112	0.063	0.068	0.674	0.538	0.423	0.031	0.128	0.529	0.601	0.585	*

*Rst values are located in the lower part of the matrix. Fst P-values were calculated from 10100 permutations and are located in the upper part of the matrix. TG – East Kazakhstan (this study), KM – Kalmyks (16), AK1 – Altaian Kazakhs (15), AK2 – Altaian Kazakhs (15), KZ1 – Kazakhs (17), KG – Kirghiz (17), UI – Uighurs (17), KZ2 – Kazakhs (18), UZ – Uzbeks (18), MG1 – Mongolians (19), MG2 – Mongolians (19) UI2 – Uighurs (19), KZ3 – Kazakhs (YA003729, *www.yhrd.com*).

### Y chromosome analysis

*Genotyping.* Sixty-seven DNA samples were amplified with the AmpFLSTR Yfiler® PCR Amplification Kit (Applied Biosystems, Warrington, UK) according to the manufacturers’ instructions. It includes 17 Y-specific STR loci (DYS456, DYS389, DYS390 23, DYS389, DYS458, DYS19, DYS385 a and b, DYS393, DYS391, DYS439, DYS635, DYS392, DYS437, DYS438, DYS448, GATA 22) that were typed in three multiplexes on an ABI3100 Genetic Analyzer (Applied Biosystems/Hitachi). A quality control check was performed by successful completing of the quality assessment exercise in 2011. After certification, the data were deposited to the Y-Chromosome Haplotype Reference Database (accession number YA003700).

*Data analyses.* Fragment sizes were determined using the GeneScan 3.1.2 software (Applied Biosystems) and allele designations were performed using the Genotyper 2.5.2 software (Applied Biosystems). We also included published data on Altaian Kazakhs, Kalmyks, Kazakhs, Kyrgyzs, Mongolians, Uighurs, and Uzbeks (Supplementary Table 2 (web extra material 2)). Haplotype diversity was calculated as described in the literature (22). Discrimination capacity was calculated as D = N_diff_/N, where N_diff_ is the number of different haplotypes of the population. Basic parameters of molecular diversity and population genetic structure, including Slatkin’s Rst matrices for pairwise genetic distances were calculated using the software package Arlequin 3.5.1.2 (University of Bern, Bern, Switzerland). The statistical significance (*P*-values) was estimated by permutation analysis, using 10100 permutations. The STATISTICA package (StatSost, Tulsa, OK, USA) was used for multidimensional scaling (MDS) analysis. Y predictor by Vadim Urasin, v.1.5.0 was used for Y-STR haplogroup prediction (*http://predictor.ydna.ru/*).

## Results

The study of mtDNA haplogroup variation in the population of East Kazakhstan region revealed that the majority of maternal lineages were distributed among haplogroups that were common in Central Asian region (Table 1).

**Figure 2:**
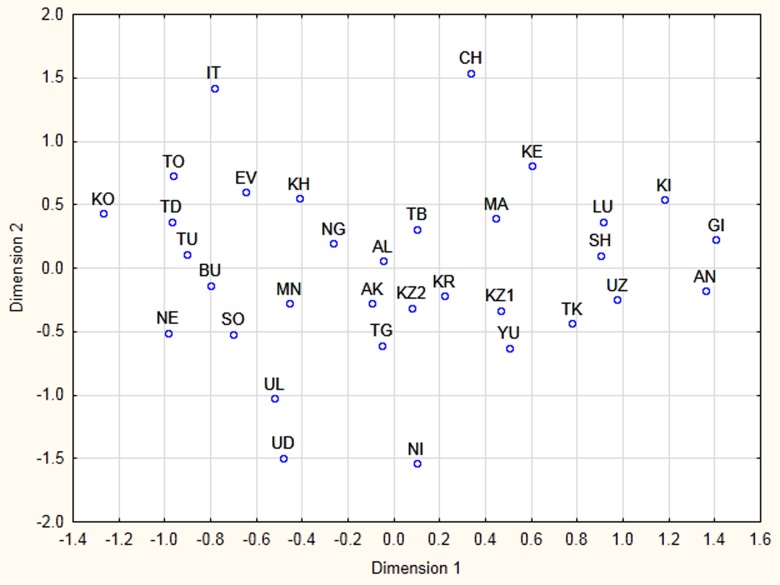
Multi-dimensional scaling plot of pair differences based on mitochondrial DNA haplogroup frequencies in Eurasian populations. The following abbreviations were used to assign populations: AK – Altaian Kazakhs (6), KZ1 – Kazakhs (1), KZ2 – Kazakhs (7), KR – Kirghiz (1), MN – Mongolians (6), UI – Uighurs (7), AL – Altaians (8), KH – Khakassians (8), BU – Buryats (8), SO – Sojots (8), TD – Todjins (8), TU – Tuvinians (8), TO – Tofalars (8), MA – Mansi (9), KE – Ket (10), NG – Nganasan (9), TB – Tubalar (11), EV – Evenki (11), NE – Negedal (11), UL – Ulchi (11), NI – Nivkhi (11), UD – Udegey (11), IT – Itelmen (12), KO – Koriak (12), CH – Chukchi (13), AN – Turks (14), GI – Gilaki (14), KI – Kurdish (14), LU – Lur (14), UZ – Uzbek (14), TK – Turkmen (14), SH – Shugnan (14), TG – Kazakhs (this study).

Comparison of 33 Eurasian populations based on genetic distances was performed and the results are presented as a multi-dimensional scaling plot (Figure 2). The populations genetically closest to the Tarbagatay population were Mongolians from Mongolia (MN), Altaian Kazakhs from South Siberia, Russia (AK), and Kazakhs from Xinjiang, China (KZ2).

In fact, all of them had equal haplogroups A, B, C, D, F, G, H, and M, suggesting that these lineages were in the common maternal gene pool from which these different lineages had emerged. However, there were some notable differences between them. For example, Kazakhs from Xinjiang had higher frequencies of West Eurasian lineages H, T, U, and Z than Mongolians, Altaian Kazakhs, or Kazakhs from East Kazakhstan. Kazakhs from East Kazakhstan had greater diversity of haplogroups present in their mtDNA gene pool than the groups from South Siberia, Xinjiang, or Mongolia. Since recent data showed a common ancestry of indigenous Altaians with Native Americans (23), we compared East Kazakhstan population with the Native Amerindians. A genetic-distance analysis indicated divergence from the Native American populations (Supplementary Table 3 (web extra material 3).

Analysis of 17 fast evolving Y-STRs provided additional details that helped to elucidate the paternal diversity in the Kazakh population. A total of 26 different haplotypes were identified in 67 individuals. Twenty-four haplotypes were nonrecurring. Paternal genetic variation within the population was rather low. Relative frequency of haplotype 15-12-29-23-10-13-12-13,18-10-12-15-19-15-17-19-12 (DYS19, DYS389I, DYS389II, DYS390, DYS391, DYS392, DYS393, DYS385a,b, DYS438, DYS439, DYS437, DYS448, DYS456, DYS458, DYS635, GATA H4) was 0.612 (Table 2). The haplotype with an identical set of alleles was also found in the population of southeastern Altaian Kazakhs, with the relative frequency of 0.214 (15). The haplotype diversity of 17 STR loci was 0.629 ± 0.071. The overall discrimination capacity was 0.388. We also reduced the 17-STR profile to a 5-STR profile (DYS389I, DYS390, DYS391, DYS392, and DYS393) to compare our population data with the published data sets. As a result of this reduction, 26 Altaian Kazakh haplotypes collapsed into 22 unique haplotypes, and the number of shared haplotypes increased.

**Figure 3:**
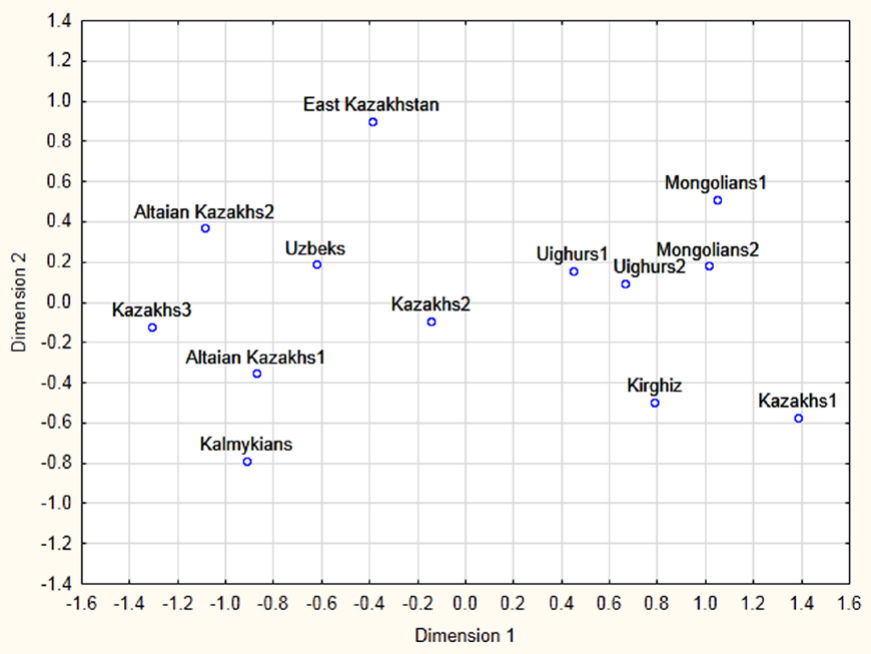
Multi-dimensional scaling plot of Rst distances based on Y-short tandem repeats (STR) haplotypes in Eurasian populations.

Rst values showed that population of East Kazakhstan remained distinctive even with a reduced number of Y-STRs. Based on the Slatkin’s Rst matrix, the populations genetically closest to the Tarbagatay population were Kazakhs from Kara-Kalpakia (Kazakhs2), southeastern Altaian Kazakhs (Altaian Kazakhs2), Uighurs from South East Kazakhstan (Uighurs1), and Uzbeks from Kara-Kalpakia (Uzbeks) (Table 3). Our population showed large genetic distances from Kyrgyzs and Kalmyks. MDS plot showed that the studied population was genetically closest to population of Uzbeks from Uzbekistan (Kara-Kalpakia) (Figure 3).

## Discussion

This study found a high degree of genetic differentiation on the level of mitochondrial DNA, but very low genetic diversity of Y-STR data.

Genetically close subpopulations of Kazakhs from Altai, Kazakhstan, and Xinjiang showed a similar mtDNA composition consisting of mainly East Eurasian haplogroups. Affinities among these populations may result from their common origin or a recent admixture resulting from geographic proximity. Genetic distances between populations can be related to geographic distances, according to a model of isolation by distance (22). Comparably high frequencies of European lineages are consistent with the intermediate position of the East Kazakhstan region in Eurasia but some inconsistent features were also present in the distribution of frequencies in mtDNA lineages. For example, relatively high frequencies of East Asian haplogroups (D, C, F, and G) in this population imply significant contribution from Siberian and Central Asian populations. These differences indicate that the population history of the studied group of Kazakhs was different from other groups.

The paternal lineages present in the Tarbagatay population were different from the populations of Kazakhs that have been studied before, with an exception of southeastern Altaian Kazakhs, who had 19 haplotypes (21.35% out of the sample size) identical with the most common haplotype observed in East Kazakhstan. This could be explained by historical data indicating that Kazakhs began migrating to the Altai region in the 19th century. According to Oktyabrskaya (24), there were 1777 people in the Kazakh Altai in 1880, 70% of them belonging to the genus Naiman and 20% to the genus Kerey.

Our results indicate that the studied subpopulation of Kazakhs has low paternal genetic diversity, and share the common paternal ancestry. The existence of a common ancestor is supported by the predominance of a single haplotype in the population. It is also supported by “*shezhire*,” which states that the Naiman tribe has a common single ancestor from Uzbekistan. These verbal genealogical data are consistent with the results of the MDS plot. An alternative explanation for our results is provided by historian Sultanov (25), who suggested that 240-360 thousand of nomads, including Naimans, migrated to the territory of modern Uzbekistan in the beginning of the 16th century. Further population studies are required to compare the genetic profiles of Naimans from Central and South-Eastern regions of Kazakhstan. In addition, the Naimans also inhabit the territory of Mongolia (Bayan-Ölgii province), Russia (Altai Republic), and China (Xinjiang Uygur Autonomous Region). According to unofficial estimates, the population of Naimans in 1917 was over 800 thousand people. Unfortunately, population studies of the Kazakh tribes are currently not being conducted.

In addition, we genotyped 99 male individuals from South Kazakhstan region populated by a different tribe (accession number YA003729, *www.yhrd.com*). Not a single 17 Y-STR haplotype was found to be common between South and East Kazakhstan region. This implies different paternal origin of the tribes and genetic substructuring among Kazakhs.

The comparison with the central “star cluster” profile, described by Zerjal et al (4), showed that only one haplotype from East Kazakhstan can possibly be assigned to the “genetic legacy of the Mongols.” It may be concluded that the influence of Genghis Khan’s Y-chromosomal lineage was insignificant in spite of the two centuries long rule of Genghis Khan and his descendants over the Naimans (mid-13th to mid-15th century) (4,26-28).

The studied subpopulation represents a genetically isolated group with a single paternal founder lineage different from the “star cluster” lineage. The presented results reveal different migration patterns in East Kazakhstan region, showing more migration among women. Partially, this paternal genetic uniformity could be explained by local traditions, such as exogamy, that were strictly followed in the past and played a crucial role in conservation of the unique genetic properties. The population of Madjar from Torgay area had been following same traditions in the past aiming to avoid inbreeding (3). Nonetheless, mtDNA genetic diversity in this population is equivalent to that in other Central Asian populations.

Our finding on active migration of maternal DNA requires additional explanation and further support from complementary sources, especially since the studied nomadic tribe is scarcely described in historical sources. Apart from additional genotyping of the Kazakh population, further cultural and historical studies are needed to gain more knowledge on the cultural processes that have greatly influenced genetic variability of this and other populations.
