# Supplementary material for: Mitochondrial and Y-chromosomal profile of the Kazakh population from East Kazakhstan
Source: Croat Med J. 2013 Feb;54(1):17–24. doi: 10.3325/cmj.2013.54.17 (PMC3583390; doi:10.3325/cmj.2013.54.17)
Supplement: Supplementary Table 1 [file CroatMedJ_54_s003.pdf]

Supplementary table 1. Characteristics of mtDNA studies in Eurasian populations

**Population**	**n***	**Location**	**Reference No.**
Altaian Kazakhs	237	Altai Republic	6
Altaians	110	Altai Republic	7
Buryats	91	South Siberia	7
Chukchi	66	Siberia	8
Evenki	71	Siberia	9
Gilaki	37	Northern Iran	10
Itelmen	47	Siberia	11
Kazakhs1	55	South Kazakhstan	1
Kazakhs2	53	Xinjiang	12
Ket	38	Northern Eurasia	13
Khakass	53	South Siberia	7
Kirghiz	95	South Kyrgyzstan	1
Koryak	155	Siberia	11
Kurd	20	Western Iran	10
Lur	17	Southwestern Iran	10
Mansi	98	Northwest Siberia	14
Mongolians	89	Mongolia	6
Negedal	33	Siberia	9
Nganasan	24	Northern Eurasia	14
Nivkh	56	Siberia	9
Shugnan	44	Tajikistan	10
Sojots	30	South Siberia	7
Tarbagatay	160	East Kazakhstan	This study
Todjins	48	South Siberia	7
Tofalars	58	South Siberia	7
Tubalar	72	Siberia	9
Turkmen	41	Turkmenistan	10
Turks	50	Anatolia, Turkey	10
Tuvinians	90	South Siberia	7
Udegey	46	Siberia	9
Uighurs	47	Xinjiang	12
Ulchi	87	Siberia	9
Uzbek	42	South Uzbekistan	10

***Abbreviation: n – number of individuals.**
