# Supplementary material for: Mitochondrial and Y-chromosomal profile of the Kazakh population from East Kazakhstan
Source: Croat Med J. 2013 Feb;54(1):17–24. doi: 10.3325/cmj.2013.54.17 (PMC3583390; doi:10.3325/cmj.2013.54.17)
Supplement: Supplementary Table 2 [file CroatMedJ_54_s004.pdf]

Supplementary table 2. Characteristics of Y-chromosome studies in Eurasian populations

**Population**	**n***	**Location**	**Reference No.**
Altaian Kazakhs1 (AK1)	30	South West Altai	15
Altaian Kazakhs2 (AK2)	89	South East Altai	15
Kalmyks (KM)	99	Kalmykia, Russia	16
Kazakhs1 (KZ1)	49	South East Kazakhstan	17
Kazakhs2 (KZ2)	50	Kara-Kalpakia	18
Kirghiz (KG)	41	North Kyrgyzstan	17
Mongolians1 (MG1)	45	Inner Mongolia, China	19
Mongolians2 (MG2)	65	Outer Mongolia	19
Tarbagatay (TG)	67	East Kazakhstan	This study
Uighurs1 (UI1)	39	South East Kazakhstan	17
Uighurs2 (UI2)	31	Urumqi	19
Uzbeks (UZ)	40	Kara-Kalpakia	18
Kazakhs3 (KZ3)	99	South Kazakhstan	Unpublished data

*__Abbreviation: n – number of individuals.__
