# Supplementary material for: Mitochondrial and Y-chromosomal profile of the Kazakh population from East Kazakhstan
Source: Croat Med J. 2013 Feb;54(1):17–24. doi: 10.3325/cmj.2013.54.17 (PMC3583390; doi:10.3325/cmj.2013.54.17)
Supplement: Supplementary Table 3 [file CroatMedJ_54_s005.pdf]

Supplementary table 3. Haplogroup frequencies in Native American populations (29)

	A (%)	B (%)	C (%)	D (%)	n
**Apache**	40.0	25.0	35.0	0.0	20
**Arsario**	63.8	0.0	36.2	0.0	47
**Cofan**	0.0	25.0	75.0	0.0	4
**Catamarca**	24.0	4.0	24.0	48.0	25
**Cayapa**	25.0	25.0	25.0	25.0	32
**Coreguaje**	3.7	18.5	66.7	11.1	27
**Dogrib**	100.0	0.0	0.0	0.0	23
**Embera**	23.1	53.8	23.1	0.0	39
**Ijka**	89.7	3.4	6.9	0.0	29
**Kuna**	61.8	38.2	0.0	0.0	34
**Kogui**	56.3	0.0	43.8	0.0	48
**Mocovi**	80.0	0.0	0.0	20.0	5
**Ngöbe**	63.6	36.4	0.0	0.0	77
**N. Paiute**	10.0	40.0	15.0	35.0	20
**Salta**	22.2	38.9	27.8	11.1	18
**Secoya**	10.0	20.0	70.0	0.0	2
**Siona**	0.0	50.0	50.0	0.0	10
**Zuni**	18.2	63.6	18.2	0.0	11
**Tucuman**	33.3	11.1	55.6	0.0	9
**Vaupes**	22.7	13.6	36.4	27.3	22
**Waunana**	31.6	36.8	26.3	5.3	57
**Wayuu**	38.1	26.2	35.7	0.0	42
